# Supplementary material for: Reconstruction of the metabolic network of Pseudomonas aeruginosa to interrogate virulence factor synthesis
Source: Nat Commun. 2017 Mar 7;8:14631. doi: 10.1038/ncomms14631 (PMC5344303; doi:10.1038/ncomms14631)
Supplement: Supplementary Information — Supplementary figures. [file ncomms14631-s1.pdf]

Supplementary Information

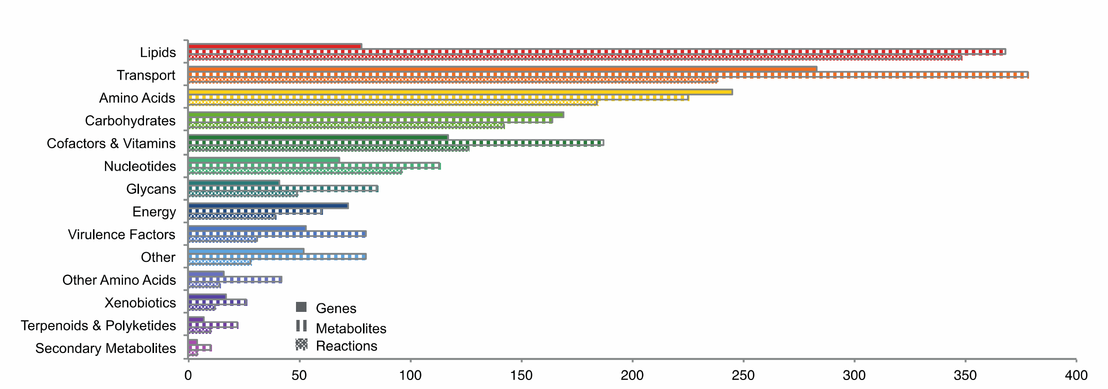

Supplementary Figure 1. Network component distribution of mPAO1. The distribution of the genes, metabolites and reactions in mPAO1 across KEGG functional categories.

A.

Metabolite ID	Carbon source name	Experimental	Computational
cpd00136	4-Hydroxy Benzoic Acid	Growth	Growth
cpd00029	Acetic Acid	Growth	Growth
cpd00137	Citric Acid	Growth	Growth
cpd00080	D,L-α-Glycerol- Phosphate	Growth	Growth
cpd00117	D-Alanine	Growth	Growth
cpd00082	D-Fructose	Growth	Growth
cpd00182	Adenosine	Growth	Growth
cpd00106	Fumaric Acid	Growth	Growth
cpd00051	L-Arginine	Growth	Growth
cpd00132	L-Asparagine	Growth	Growth
cpd00041	L-Aspartic Acid	Growth	Growth
cpd00023	L-Glutamic Acid	Growth	Growth
cpd00053	L-Glutamine	Growth	Growth
cpd00119	L-Histidine	Growth	Growth
cpd00100	Glycerol	Growth	Growth
cpd00033	Glycine	Growth	Growth
cpd00380	Itaconic Acid	Growth	Growth
cpd00064	L-Ornithine	Growth	Growth
cpd00066	L-Phenylalanine	Growth	Growth
cpd00129	L-Proline	Growth	Growth
cpd00054	L-Serine	Growth	Growth
cpd00308	Malonic Acid	Growth	Growth
cpd00035	L-Alanine	Growth	Growth
cpd00027	α-D-Glucose	Growth	Growth
cpd00118	Putrescine	Growth	Growth
cpd00020	Pyruvic Acid	Growth	Growth
cpd00036	Succinic Acid	Growth	Growth
cpd00024	α-Keto-Glutaric Acid	Growth	Growth
cpd00281	γ-Amino Butyric Acid	Growth	Growth
cpd00322	L-Isoleucine	Growth	Growth
cpd00130	L-Malic Acid	Growth	Growth
cpd00159	L-Lactic Acid	Growth	Growth
cpd00107	L-Leucine	Growth	Growth
cpd00314	D-Mannitol	Growth	Growth
cpd00162	2-Aminoethanol	Growth	Growth

Metabolite ID	Carbon source name	Experimental	Computational
cpd01949	2,3-Butanediol	No Growth	No Growth
cpd00361	2,3-Butanone	No Growth	No Growth
cpd00158	D-Cellobiose	No Growth	No Growth
cpd00072	D-Fructose-6-Phosphate	No Growth	No Growth
cpd00280	D-Galacturonic Acid	No Growth	No Growth
cpd00089	D-Glucose-1-Phosphate	No Growth	No Growth
cpd00079	D-Glucose-6-Phosphate	No Growth	No Growth
cpd00609	D-Saccharic Acid	No Growth	No Growth
cpd00138	D-Mannose	No Growth	No Growth
cpd00164	D-Glucuronic Acid	No Growth	No Growth
cpd00588	D-Sorbitol	No Growth	No Growth
cpd00666	D-Tartaric Acid	No Growth	No Growth
cpd00154	D-Xylose	No Growth	No Growth
cpd00047	Formic Acid	No Growth	No Growth
cpd00155	Glycogen	No Growth	No Growth
cpd00139	Glycolic Acid	No Growth	No Growth
cpd00040	Glyoxylic Acid	No Growth	No Growth
cpd11589	Glycyl-L-Aspartic Acid	No Growth	No Growth
cpd00294	2-Deoxy Adenosine	No Growth	No Growth
cpd00060	L-Methionine	No Growth	No Growth
cpd00666	L-Tartaric Acid	No Growth	No Growth
cpd01242	2-Deoxy-D-Ribose	No Growth	No Growth
cpd00184	Thymidine	No Growth	No Growth
cpd00794	D-Trehalose	No Growth	No Growth
cpd00156	L-Valine	No Growth	No Growth
cpd00179	Maltose	No Growth	No Growth
cpd01262	Maltotriose	No Growth	No Growth
cpd03320	m-Hydroxy Phenyl Acetic Acid	No Growth	No Growth
cpd00094	α-Keto-Butyric Acid	No Growth	No Growth
cpd00121	m-Inositol	No Growth	No Growth
cpd00666	m-Tartaric Acid	No Growth	No Growth
cpd00652	Mucic Acid	No Growth	No Growth
cpd00224	L-Arabinose	No Growth	No Growth
cpd00227	L-Homoserine	No Growth	No Growth
cpd00599	2-Hydroxy Benzoic Acid	No Growth	No Growth
cpd00361	3-Hydroxy 2-Butanone	No Growth	No Growth
cpd01502	Citraconic Acid	No Growth	No Growth
cpd00142	Acetoacetic Acid	No Growth	No Growth

Metabolite ID	Carbon source name	Experimental	Computational
cpd00386	D-Malic Acid	No Growth	Growth
cpd00105	D-Ribose	No Growth	Growth
cpd00550	D-Serine	No Growth	Growth
cpd00246	Inosine	No Growth	Growth
cpd00161	L-Threonine	No Growth	Growth
cpd00477	N-Acetyl-L-Glutamic Acid	Growth	No Growth
cpd11592	Glycyl-L-Glutamic Acid	Growth	No Growth
cpd11588	Glycyl-L-Proline	Growth	No Growth
cpd00851	Hydroxy-L-Proline	Growth	No Growth
cpd00211	Butyric Acid	Growth	No Growth
cpd11585	L-Alanyl-Glycine	Growth	No Growth
cpd00039	L-Lysine	Growth	No Growth
cpd00266	D,L-Carnitine	Growth	No Growth
cpd00222	D-Gluconic Acid	Growth	No Growth
cpd00489	p-Hydroxy Phenyl Acetic Acid	Growth	No Growth
cpd00141	Propionic Acid	Growth	No Growth
cpd00249	Uridine	Growth	No Growth
cpd00797	β-Hydroxy Butyric Acid	Growth	No Growth

Growth
No Growth

Supplementary Figure 2. A) Comparison of experimental and computational single substrate source utilization for *P. aeruginosa* PA14. B) Comparison of experimental and computational single substrate source utilization for *P. aeruginosa* PAO1.

B.

Metabolite ID	Carbon source name	Experimental	Computational
cpd00162	2-Aminoethanol	Growth	Growth
cpd00136	4-Hydroxy Benzoic Acid	Growth	Growth
cpd00029	Acetic Acid	Growth	Growth
cpd00331	cis-Aconitic Acid	Growth	Growth
cpd00137	Citric Acid	Growth	Growth
cpd00117	D-Alanine	Growth	Growth
cpd00082	D-Fructose	Growth	Growth
cpd00314	D-Mannitol	Growth	Growth
cpd00106	Fumaric Acid	Growth	Growth
cpd00100	Glycerol	Growth	Growth
cpd00246	Inosine	Growth	Growth
cpd00380	Itaconic Acid	Growth	Growth
cpd00035	L-Alanine	Growth	Growth
cpd00051	L-Arginine	Growth	Growth
cpd00132	L-Asparagine	Growth	Growth
cpd00041	L-Aspartic Acid	Growth	Growth
cpd00023	L-Glutamic Acid	Growth	Growth
cpd00053	L-Glutamine	Growth	Growth
cpd00119	L-Histidine	Growth	Growth
cpd00322	L-Isoleucine	Growth	Growth
cpd00159	L-Lactic Acid	Growth	Growth
cpd00107	L-Leucine	Growth	Growth
cpd00130	L-Malic Acid	Growth	Growth
cpd00064	L-Ornithine	Growth	Growth
cpd00066	L-Phenylalanine	Growth	Growth
cpd00129	L-Proline	Growth	Growth
cpd00054	L-Serine	Growth	Growth
cpd00308	Malonic Acid	Growth	Growth
cpd00118	Putrescine	Growth	Growth
cpd00020	Pyruvic Acid	Growth	Growth
cpd00036	Succinic Acid	Growth	Growth
cpd00581	Urocanic Acid	Growth	Growth
cpd00027	α-D-Glucose	Growth	Growth
cpd00024	α-Keto-Glutaric Acid	Growth	Growth
cpd00281	γ-Amino Butyric Acid	Growth	Growth

Metabolite ID	Carbon source name	Experimental	Computational
cpd01949	2,3-Butanediol	No Growth	No Growth
cpd00361	2,3-Butanone	No Growth	No Growth
cpd00294	2-Deoxy Adenosine	No Growth	No Growth
cpd01242	2-Deoxy-D-Ribose	No Growth	No Growth
cpd00599	2-Hydroxy Benzoic Acid	No Growth	No Growth
cpd00361	3-Hydroxy 2-Butanone	No Growth	No Growth
cpd00142	Acetoacetic Acid	No Growth	No Growth
cpd01502	Citraconic Acid	No Growth	No Growth
cpd00158	D-Cellobiose	No Growth	No Growth
cpd00072	D-Fructose-6-Phosphate	No Growth	No Growth
cpd00280	D-Galacturonic Acid	No Growth	No Growth
cpd00089	D-Glucose-1-Phosphate	No Growth	No Growth
cpd00079	D-Glucose-6-Phosphate	No Growth	No Growth
cpd00164	D-Glucuronic Acid	No Growth	No Growth
cpd00138	D-Mannose	No Growth	No Growth
cpd00609	D-Saccharic Acid	No Growth	No Growth
cpd00588	D-Sorbitol	No Growth	No Growth
cpd00666	D-Tartaric Acid	No Growth	No Growth
cpd00794	D-Trehalose	No Growth	No Growth
cpd00154	D-Xylose	No Growth	No Growth
cpd00047	Formic Acid	No Growth	No Growth
cpd00155	Glycogen	No Growth	No Growth
cpd00139	Glycolic Acid	No Growth	No Growth
cpd11589	Glycyl-L-Aspartic Acid	No Growth	No Growth
cpd11592	Glycyl-L-Glutamic Acid	No Growth	No Growth
cpd00040	Glyoxylic Acid	No Growth	No Growth
cpd11585	L-Alanyl-Glycine	No Growth	No Growth
cpd00224	L-Arabinose	No Growth	No Growth
cpd00227	L-Homoserine	No Growth	No Growth
cpd00060	L-Methionine	No Growth	No Growth
cpd00666	L-Tartaric Acid	No Growth	No Growth
cpd00179	Maltose	No Growth	No Growth
cpd01262	Maltotriose	No Growth	No Growth
cpd03320	m-Hydroxy Phenyl Acetic Acid	No Growth	No Growth
cpd00121	m-Inositol	No Growth	No Growth
cpd00666	m-Tartaric Acid	No Growth	No Growth
cpd00652	Mucic Acid	No Growth	No Growth
cpd00184	Thymidine	No Growth	No Growth
cpd00249	Uridine	No Growth	No Growth
cpd00094	α-Keto-Butyric Acid	No Growth	No Growth

Metabolite ID	Carbon source name	Experimental	Computational
cpd00182	Adenosine	No Growth	Growth
cpd00080	D,L-α-Glycerol- Phosphate	No Growth	Growth
cpd00386	D-Malic Acid	No Growth	Growth
cpd00105	D-Ribose	No Growth	Growth
cpd00550	D-Serine	No Growth	Growth
cpd00033	Glycine	No Growth	Growth
cpd00161	L-Threonine	No Growth	Growth
cpd00211	Butyric Acid	Growth	No Growth
cpd00266	D,L-Carnitine	Growth	No Growth
cpd00222	D-Gluconic Acid	Growth	No Growth
cpd11588	Glycyl-L-Proline	Growth	No Growth
cpd00851	Hydroxy-L-Proline	Growth	No Growth
cpd00039	L-Lysine	Growth	No Growth
cpd00156	L-Valine	Growth	No Growth
cpd00477	N-Acetyl-L-Glutamic Acid	Growth	No Growth
cpd00489	p-Hydroxy Phenyl Acetic Acid	Growth	No Growth
cpd00141	Propionic Acid	Growth	No Growth
cpd00797	β-Hydroxy Butyric Acid	Growth	No Growth

Growth
No Growth

Supplementary Figure 2. A) Comparison of experimental and computational single substrate source utilization for *P. aeruginosa* PA14. B) Comparison of experimental and computational single substrate source utilization for *P. aeruginosa* PAO1.

A.

in vitro

in silico		+ 95	- 1037	
+	110	True Positive 54	False Positive 56	Positive Predictive Value 49.09%
-	1022	False Negative 41	True Negative 981	Negative Predictive Value 95.99%
		Sensitivity 56.84%	Specificity 94.60%	Accuracy 91.43%

B.

in vitro

in silico		+ 96	- 1052	
+	110	True Positive 54	False Positive 56	Positive Predictive Value 49.09%
-	1038	False Negative 42	True Negative 996	Negative Predictive Value 95.95%
		Sensitivity 56.25%	Specificity 94.68%	Accuracy 91.46%

Supplementary Figure 3. Analysis of essential gene predictions compared to *in vitro* essentiality. Overlap of essential genes derived from the PAO1 single transposon mutant library and PA14 single transposon mutant library were used. Both mPA14 (Panel A) and mPAO1 (Panel B) predict gene essentiality with an accuracy of 91%.
